# Supplementary material for: Single-molecule insights into surface-mediated homochirality in hierarchical peptide assembly
Source: Nat Commun. 2018 Jul 13;9:2711. doi: 10.1038/s41467-018-05218-0 (PMC6045617; doi:10.1038/s41467-018-05218-0)
Supplement: Supplementary file 1 — Supplementary Information [file 41467_2018_5218_MOESM1_ESM.pdf]

Supplementary information

Chen et al. Single-molecule Insights into Surface-mediated Homochirality in Hierarchical Peptide Assembly

1. Supplementary Figures

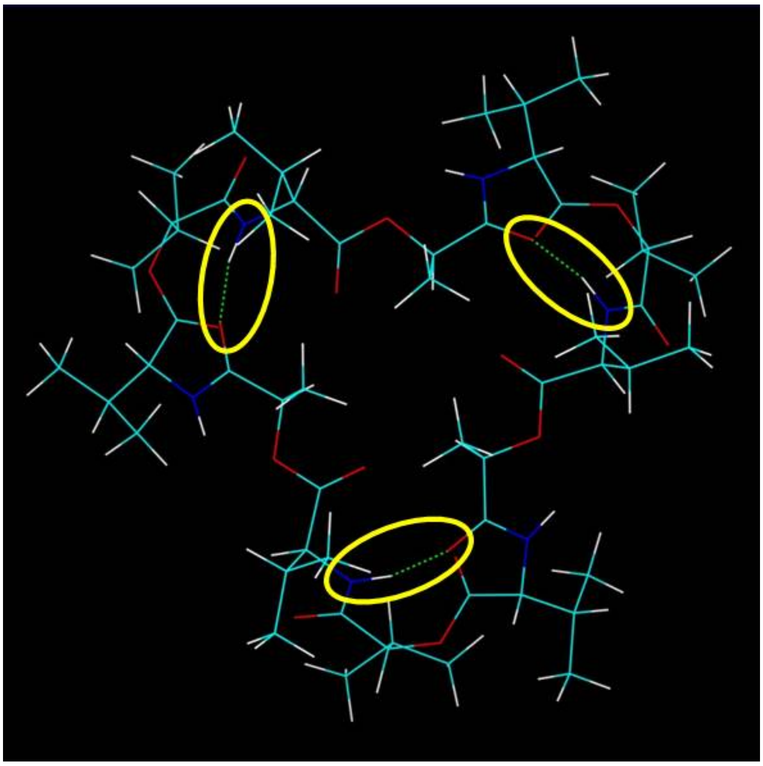

Supplementary Figure 1. Calculated molecular model of valinomycin viewed perpendicular to the A face. In DFT calculated molecular model, carbon, nitrogen, oxygen and hydrogen atoms are displayed in cyan, blue, red and gray, respectively. Three intramolecular N-H•••O hydrogen bonds form between the carbonyl oxygen of L-Lac residue and the amine group of D-Val residue which are marked by yellow circles. H•••O are shown by green dotted lines.

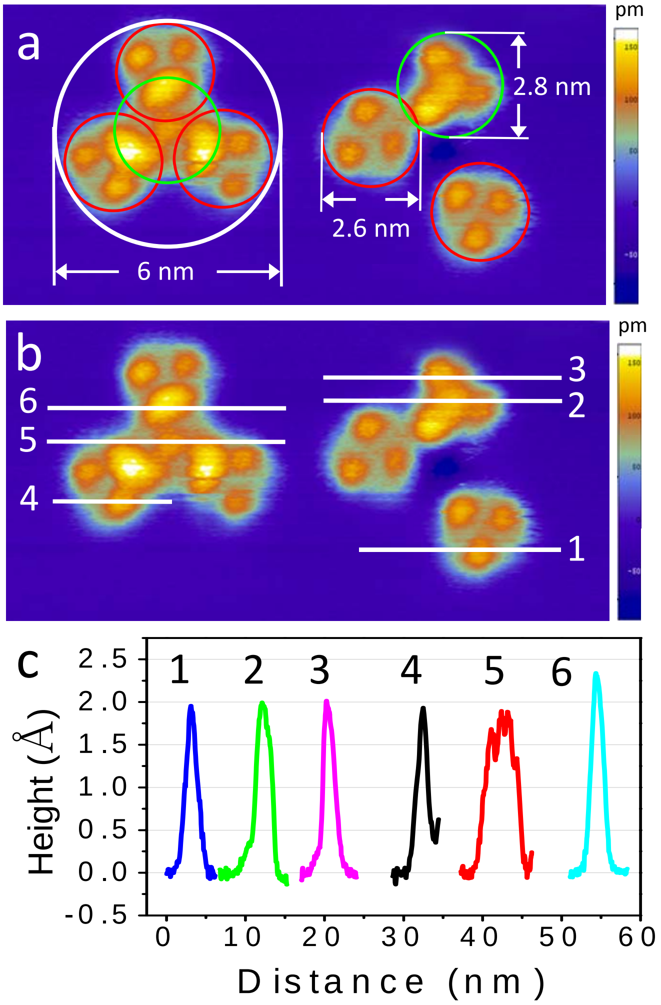

Supplementary Figure 2. Comparison between valinomycin tetramer and monomers. a, b. STM images (16 nm x 8 nm) of valinomycin tetramer coexists with monomers (M_L & M_R). c. The cross sections for the valinomycin in Figure 2b.

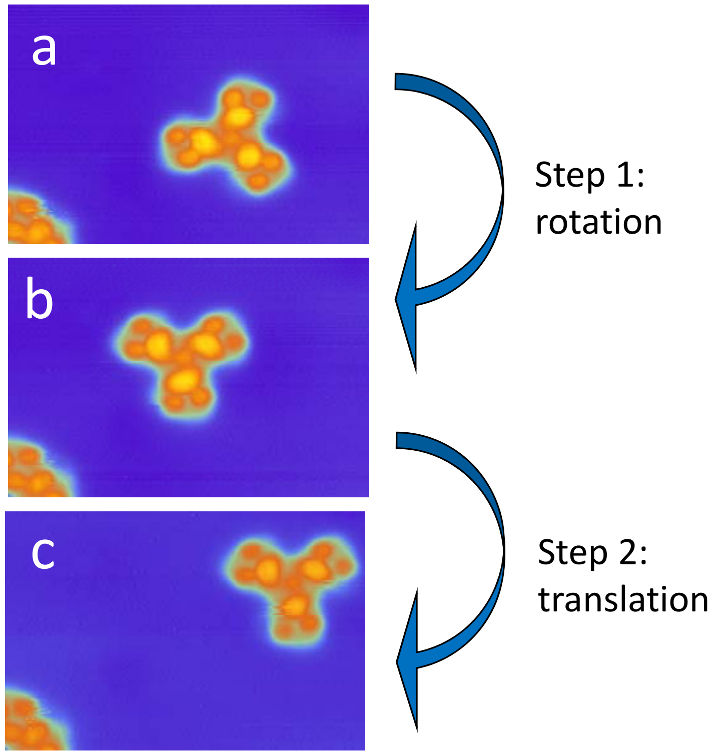

Supplementary Figure 3. The rotation and translation manipulation of valinomycin tetramer. The rotation manipulation process was from Figure 3a to 3b. The translation process was from Figure 3b to 3c. The size of STM images is 15 nm x 10 nm.

2. Supplementary Table

Supplementary Table 1. Calculated interaction energies of valinomycin tetramer on the Cu(111) surface by DFT-D3 methods. A more negative energy indicates that the calculated system is more stable.

Tetramer model	Sum of interaction energy among monomer subunits (kcal·mol^{-1})	Tetramer-substrate interaction energy (kcal·mol^{-1})	Total interaction energy (kcal·mol^{-1})
M_L-3M_R-right	-43.127	-249.977	-293.104
M_L-3M_R-left	-31.868	-251.309	-283.177
M_R-3M_L-right	-34.777	-237.708	-272.485
M_R-3M_L-left	-33.823	-238.064	-271.887
M_R-3M_R-right	-27.521	-264.256	-291.777
M_R-3M_R-left	-24.117	-256.112	-280.229
M_L-3M_L-right	-26.856	-228.394	-255.250
M_L-3M_L-left	-23.897	-215.878	-239.775
